# Bacterial Production of Recombinant Coagulation Factor VIII Domains

**DOI:** 10.3390/medicina59040694

**Published:** 2023-04-01

**Authors:** Saima Bashar, Hee-Jin Jeong

**Affiliations:** 1 Industry-Academia Cooperation Foundation, Hongik University, 2639 Sejong-ro, Sejong-si 30016, Republic of Korea; 2 Department of Biological and Chemical Engineering, Hongik University, 2639 Sejong-ro, Sejong-si 30016, Republic of Korea

**Keywords:** coagulation factor disorders, factor VIII, recombinant protein, hemophilia A

## Abstract

Factor VIII (F8) is a blood coagulation protein prearranged in six domains, and its deficiency causes hemophilia A. To fashion functional F8 therapeutics, development of a recombinant F8 (rF8) domain is essential not only for F8 substitution, but also to decipher the F8-related mechanisms. In this study, we generated Glutathione S-transferase (GST)-conjugated recombinant A2 and A3 domains of F8 using *Escherichia coli*. The high growth rate and economically advantageous protein production system in terms of inexpensive reagents and materials in *E. coli* cells facilitated the completion of entire process from protein expression to purification in 3–4 days with low production cost. Subsequent assessment of these purified proteins using enzyme-linked immunosorbent assay (ELISA) and antibodies against F8 revealed enhanced detection of rF8-A2 or rF8-A3 in a concentration dependent manner, indicating the presence of the antibody-binding epitopes in these proteins. Furthermore, these proteins are suitable for generating novel antibodies against the F8 domain and F8 domain-capturing affinity columns by enabling their conjugation to GST-capturing beads. Additionally, the recombinant F8 domains produced herein can be used for various studies, which include investigating the explicit roles of the F8 domain in the coagulation process, with domain-specific binding partners, and antibodies.

## 1. Introduction

Hemophilia A is a congenital bleeding disorder caused by factor VIII (F8) deficiency. F8 comprises of multiple domains designated and arranged in the order A1-A2-B-A3-C1-C2. A full-length F8 molecule is divided into a heavy chain (A1-A2-B) and a light chain (A3-C1-C2). In addition, the A1 and A3 domains of these chains are linked by metal ion-mediated non-covalent bonding in the circulating blood [1,2]. These chains are activated by thrombin and circulate as heterotrimer consisting of A1, A2, and A3-C1-C2, after releasing the B domain. Although the functionality of each domain has not yet been completely characterized, it is known that the A2 domain is required for cofactor activity [3], while B domain remains unaffected by F8 activity [4,5]. Injection of F8 obtained from donated blood plasma into Hemophilia A patients has been traditionally used to address the F8 deficiency. However, the approach faces multiple challenges, such as limited supply, and potential virus and prion transmission risks [6,7,8]. Alternatively, F8 replacement therapy using recombinant F8 (rF8) has been used [9]. However, an antibody is generated against the injected rF8 during treatment, which acts as an inhibitor that blocks rF8, resulting in reduced rF8 function. However, details of the inhibitory mechanisms, such as key residues involved in antibody-binding, have not been fully elucidated. Evaluation of rF8 domains and their corresponding antibodies can affect various fundamental studies on maintaining rF8 domain functions and decreasing inhibitor binding affinity, which can eventually minimize the immunogenic response in patients. Thus, generating rF8 domains is of significant interest, and can provide valuable insights to understand and design engineered F8 therapeutics.

Mammalian cells, such as Chinese hamster ovary (CHO) cells and baby hamster kidney (BHK) cells, and bacteria, such as *Escherichia coli*, have been used as hosts to produce rF8 [2,10,11,12,13]. *E. coli*-based protein expression system have merits, such as lower manufacturing cost and higher cell growth rate than the eukaryotic protein synthesis system. Choi et al. identified DNA sequences encoding the F8 A1, A2, A3, and C domains, and subcloned these fragments into an *E. coli*-expression vector to express each protein domain in a soluble form [11]. However, the interaction between the expressed domains and the anti-F8 antibody was not addressed in their study because they focused on the characteristic validation of the factor IX-specific binding of the rF8 domain.

In this study, we aimed to produce recombinant A2 and A3 domains of F8, rF8-A2, and rF8-A3, which can be used as recombinant proteins for studying F8 domain-specific functions during blood coagulation.

## 2. Materials and Methods

Synthesized rF8-A2-and rF8-A3-encoding genes (Lncbio, Seoul, Republic of Korea) and *E. coli* expressing vector pGEX-4T1 (Addgene, Watertown, MA, USA) were amplified via PCR using Ex-premier DNA polymerase (Takara, Nojihigashi, Shiga, Japan) and primers (Table 1). The PCR products were ligated using In-Fusion HD cloning kit (Takara, Nojihigashi, Shiga, Japan). Colony PCR was performed to select the successfully ligated clones and plasmids were extracted from the selected colonies. The nucletide sequences of the plasmids were confirmed via Sanger sequencing, resulting in pGEX-4T1::rF8-A2 and pGEX-4T1::rF8-A3.

rF8-A2 and rF8-A3 proteins were expressed using SHuffle T7 lysY *E. coli* by cultivating the cells at 37 °C in 2 × YTA medium containing 0.2% glucose until OD600 reached to 1.0–1.5. After adding 0.5 mM IPTG, the cells were further incubated for 20 h at 16 °C. After sonication using lysis buffer (50 mM potassium phosphate (pH 7.0), 300 mM sodium chloride (NaCl)) with 10 mM imidazole, the supernatant was separated by centrifugation (13,000 rpm, 30 min, 4 °C). Protein purification was performed using Ni-NTA beads (GE healthcare, Chicago, IL, USA), washing buffer (lysis buffer with 20 mM imidazole) and elution buffer (lysis buffer with 500 mM imidazole) according to the manufacturer’s protocol. After exchanging the eluent buffer to phosphate-buffered saline (PBS) using 3 k MWCO ultra-filtration column (Pall, Ann Arbor, MI, USA), the concentration of protein was measured via SDS-PAGE analysis using bovine serum albumin (BSA) standards. Protein purity was calculated using ImageLab software (Version 6.1, Bio-Rad, Hercules, CA, USA).

The antibody-binding activity of purified A2 or A3 antigen was confirmed by indirect ELISA. The 96-well plate (Medi-binding, SPL, Pocheon, Gyeonggi-do, Republic of Korea) was coated with 100 μL of series diluted rF8-A2 or rF8-A3 for 16 h at 4 °C. The plate was blocked with PBS0.05T2B (PBS containing 0.05% Tween-20 and 2% BSA) for 2 h at room temperature (RT). After washing using PBS0.05T 3 times, 2000-times diluted rabbit anti-F8 polyclonal antibody (Sino Biological, Beijing, China) or 2000-times diluted mouse anti-F8-A2 monoclonal antibody (Novus Biologicals, Englewood, CO, USA) in PBS was added as a primary antibody and incubated for 1 h at RT. After washing thrice using PBS0.05T, 2000-times diluted HRP-conjugated goat anti-mouse Fc antibody (AbFrontier, Seoul, Republic of Korea) or 20,000-times diluted HRP-conjugated goat anti-rabbit IgG antibody (GW Biotech, Seoul, Republic of Korea) was added as a secondary antibody, and incubated for 1 h at RT. After thrice washing using PBST0.05T, TMB solution (TCI, Tokyo, Japan) was added. After incubation for 25 min, the reaction was stopped by adding 1 N sulfuric acid, and the absorbance at 450 nm was measured using a microplate reader. Dose–response curves were constructed by fitting the absorbance using the GraphPad Prism (Version 9, GraphPad Software, San Diego, CA, USA). The dose–response curve were fitted to a 4-parameter logistic equation of Y = Bottom + (Top − Bottom)/(1 + 10 ((LogEC50-X) × Hill Slope)); where Bottom and Top indicate absorbance in the absence of antigen and absorbance in the presence of maximum concentration of antigen, respectively. The limit of detection (LOD) value (EC10) was calculated using GraphPad Prism.

## 3. Results

Based on the human F8 gene sequence (NM_000132), we synthesized A2 (387–587 residues of F8)- and A3 (1796–2004 residues of F8)-encoding genes, which contain major epitopes of each domain [11,14,15]. The main antibody epitope of the A2 domain is present in the residues 484–508, while the epitope related to factor IXa that affects the cofactor catalyst, and the activation of the enzyme reaction is placed at 558–565 residues [2]. Likewise, for A3, it was confirmed that the factor IXa-binding sites were located on 1793–1795 and 1811–1818 residues, respectively [15]. Moreover, a previous study had described the expression of soluble A2 domain with 387–587 residues and A3 domain with 1796–2004 residues using *E. coli* [11]. Consequently, we also used these partial sequences to express the rF8-A2 and rF8-A3.

**Figure 1:**
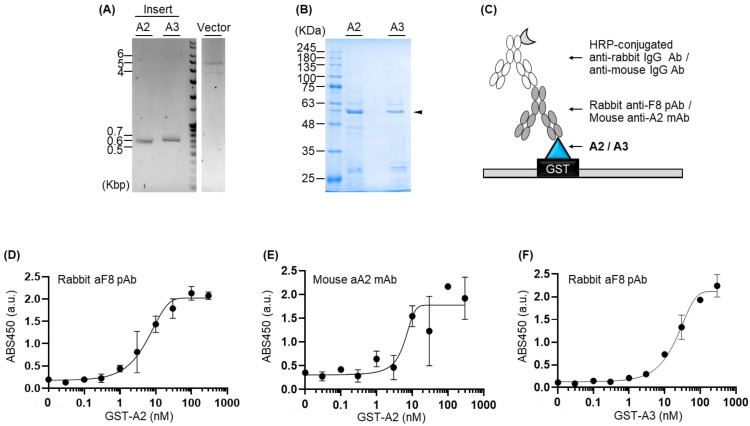
(**A**) Agarose gel electrophoresis analysis of DNA products after amplifying insert DNA (rF8-A2-or rF8-A3-encoding gene) and vector DNA (linearized pGEX-4T1); (**B**) SDS-PAGE analysis of rF8-A2 or rF8-A3. The black arrow indicates the target protein; (**C**) Schematic representation of the ELISA; (**D**) ELISA signal obtained from rF8-A2 with rabbit anti-F8 polyclonal antibody; (**E**) ELISA signal obtained from rF8-A2 with mouse anti-A2 monoclonal antibody; (**F**) ELISA signal obtained from rF8-A3 with rabbit anti-F8 polyclonal antibody. Error bars represent ±1 SD (*n* = 3). SD = standard deviation. GST, HRP, IgG, ABS, pAb, and mAb indicates glutathione S-transferase, horseradish peroxidase, immunoglobulin G, absorbance, polyclonal antibody, and monoclonal antibody, respectively.

Subsequently, we amplified the A2- or A3-encoding genes with 15 bp extensions complementary to pGEX-4T1 vector ends and linearized the vector using PCR (Figure 1A). The two PCR products were ligated using In-fusion enzyme by overlapping the extended nucleotides. Additionally, we incorporated a Glutathione S-transferase (GST) fusion partner at the N-terminus of the vector to improve the yield of the soluble protein and a His-tag at the C-terminus of the vector to facilitate the purification of the target protein from the cell lysate. *E. coli* cells were then transformed with each plasmid and induced to express protein, using IPTG. Subsequently, the target proteins were purified from the cell lysate using Ni-NTA affinity beads. Successive analysis of the purified proteins confirmed the presence of GST-tagged rF8-A2 and rF8-A3 with the expected sizes of 50.5 kDa and 51.6 kDa, respectively, in soluble form (Figure 1B, Supplementary Figure S1). In a previous study reporting the production of rF8 domains using *E. coli* in soluble form [11], we had initially produced the rF8 domains using the pET28a (+) vector. However, the yield of soluble proteins remained unsatisfactory even after optimization of the expression and induction conditions, including temperature, time, and inducer concentration (Supplementary Figure S2). To increase the yield of recombinant protein, we introduced a solubility-enhancing fusion partner sequence that encodes GST to the 5′ of F8 domain-encoding sequence by switching the vector from pET28a (+) to pGEX-4T1. As a result, the yields of the purified rF8-A2 and rF8-A3 in a 100 mL scale of production were 300–400 µg, which were higher than those of previously produced proteins using pET28a (+) vector. The purity of each protein was estimated as 74% for rF8-A2 and 52% for rF8-A3 by computing the ratio of the intensity between the target protein and total bands on the SDS-PAGE gel. We performed a His-tag rather than GST-based purification by considering the production yield and cost [16,17] and continued to confirm the antibody-binding using these proteins. This is because the presence of non-target proteins would not affect the antibody-binding owing to its high specificity for the target antigen. We further assumed that even if the primary or secondary antibody binds non-specifically to the non-target proteins, only the background signal will be slightly increased, but the confirmation of antigen-antibody activity will not be affected significantly. Nevertheless, size-exclusion chromatography or affinity purification using GST-binding beads can be additionally performed if a higher purity is required for further experimental applications using these recombinant proteins.

Furthermore, indirect ELISA was performed by immobilizing the purified rF8-A2 or rF8-A3 to the wells of a 96-well plate, followed by blocking, antibody binding, and enzymatic reaction (Figure 1C). When we used serially diluted rF8-A2 proteins as antigens, rabbit anti-F8 polyclonal antibody as the primary antibody, and anti-rabbit IgG antibody as the secondary antibody, a significant antigen-concentration-dependent signal was detected (Figure 1D). Moreover, when we performed ELISA using commercially available A2-specific monoclonal IgG2a, which was generated from mouse as a primary antibody and an anti-mouse IgG antibody as a secondary antibody, the ELISA titer was elevated (Figure 1E). The EC50 and LOD values for the titration curve from the anti-F8 antibody-based ELISA were 6.14 ± 2.60 nM and 0.95 ± 0.53 nM, respectively, and those from the anti-A2-antibody-based ELISA were 111 ± 179 nM and 2.14 ± 1.12 nM, respectively (Table 2). These results demonstrate that rF8-A2 possesses both anti-A2 antibody and anti-F8 antibody binding epitopes. Although we could not perform ELISA using a monoclonal antibody against the A3 domain owing to the absence of commercially available anti-A3 antibody, we did perform ELISA using anti-F8 polyclonal antibody as a primary antibody and rF8-A3 as an antigen. The results of the assay demonstrated an increase in the antigen titer in an antigen-concentration-dependent manner with the EC50 and LOD values being 29.7 ± 10.4 nM and 3.54 ± 0.54 nM, respectively (Figure 1F, Table 2). Commercially available anti-F8 polyclonal antibody was generated by immunizing rabbits with recombinant GST-Schistosoma japonicum and purified via protein A and human F8 affinity chromatography. As polyclonal antibodies are composed of several antibodies that bind to target antigens with different epitopes, our results using the anti-F8 polyclonal antibodies showed high binding efficiency to rF8-A3 and rF8-A2 indicating that rF8-A3 generated in this study also possesses the epitopes for interacting with anti-F8 antibody. Overall, these findings suggest that rF8-A2 and rF8-A3 have the potential to be used for various applications, such as generating a recombinant antibody against A2 or A3, and screening the antibodies that recognize F8 or A2/3 domains in the blood of hemophilia A patients with high selectivity and sensitivity.

## 4. Discussion

In this study, we describe a method for generating GST-conjugated rF8-A2 and rF8-A3 using in a soluble using *E. coli* within a short duration of 3–4 days. We confirmed that these proteins have antibody-binding properties, presenting the possibility of their medical and pharmaceutical applications, such as the screening and detection of F8- or F8 domain-specific antibodies in hemophilia patients. To date, there exist recombinant full-length F8s that can be used for research or FDA approval, and several studies reporting the production of rF8 have been published. However, to the best of our knowledge, there are only two papers that describe the production of rF8 domains. One of these papers reports the production of rA2 using insect cells and its activity confirmation via ELISA [3], while another describes the production of GST- or His-tag conjugated A1, A2, A2, and C domains using *E. coli* [11]. However, none of them report confirmation of their antibody-binding property. Therefore, the methods and results described herein will provide fundamental technological information for researchers striving to construct functional rF8 domains. Moreover, as the rF8-A2 and rF8-A3 developed in this study contain a GST-tag, these recombinant proteins may be used to develop affinity column for capturing recombinant or native F8 in human blood by conjugating these proteins to GST-tag affinity beads. In particular, the production of rF8 domains using prokaryotic expression system rather than the eukaryotes-based system allows significant decrease in the production costs, shortened production time, and more convenience of procedure without the risk of frequent contaminations.

In this study, we simultaneously constructed the rF8-A1- and rF8-C-expressing plasmids with rF8-A2- and rF8-A3-expressing plasmids to generate rF8-A1 (1796–2004 residues of F8) and rF8-C (2173–2307 residues of F8). However, these proteins were typically expressed as insoluble forms; thus, sufficient amounts of soluble proteins for ELISA were not obtained. Further studies on the optimization for expressing soluble forms of rF8-A1 and rF8-C are ongoing with the expectation of developing a tool set to develop new assays for evaluating the performance of F8 domains and anti-F8 domain antibodies. We believe that the rF8 domain panel can significantly support basic research demonstrating the efficacy of F8 related molecules. Consequently, we are planning to generate A2- A3-specific recombinant antibodies using rF8-A2 and rF8-A3 for providing stronger evidence to facilitate a comprehensive understanding of the F8 associated mechanisms.

## Figures and Tables

**Table 1:** Primers used in this study.

	Primer Name	Sequence (5′-3′)
rF8-A2	F8A2_pGEX4T1_Infulns_Fw	gttccgcgtggatccgctgctgaagaggaggac
	F8A2_pGEX4T1_Infulns_Rv	ggcagatcgtcagtcagtggtggtggtggtggtgga
rF8-A3	F8A3_pGEX4T1_Infulns_Fw	gttccgcgtggatcccagaggcaaggagcagaac
	F8A1A3_pGEX4T1_Infulns_Rv	ggcagatcgtcagtcagtggtggtggtggtggtgc
Vector	pGEX4T1_infuVec_Fw	gactgacgatctgcctcgc
	pGEX4T1_infuVec_Rv	ggatccacgcggaaccaga

**Table 2:** EC50 and LOD values from the titration curves.

Antigen	Primary Ab	EC50 (nM)			LOD (nM)		
		1st Trial	2nd Trial	3rd Trial	1st Trial	2nd Trial	3rd Trial
rF8-A2	Anti-F8 pAb	3.30	8.41	6.70	0.45	1.5	0.90
rF8-A2	Anti-A2 mAb	318	8.27	6.29	3.01	1.09	3.32
rF8-A3	Anti-F8 pAb	35.9	35.7	29.7	3.67	4.01	2.95

## Data Availability

The data presented in this study are available on request from the corresponding author.

## Supplementary Materials

The following supporting information can be downloaded at: https://www.mdpi.com/article/10.3390/medicina59040694/s1, Figure S1: (A) SDS-PAGE analysis of GST-conjugated rF8-A2 or rF8-A3 with various concentrations of bovine serum albumin (BSA). 10 μL of recombinant proteins and BSA proteins were loaded. (B) Standard curve for estimating the absolute quantity of target protein. It was calculated that 16.6 μg of GST-A2 and 9.6 μg of GST-A3 were applied on the gel for SDS-PAGE analysis.; Figure S2: SDS-PAGE analysis of the expressed pET28(a)::rF8-A2 or pET28(a)::rF8-A3 proteins. S, FT, W, and E indicate soluble fraction prior to purification, flow through fraction after Ni-NTA bead binding, washed fraction, and eluted fraction, respectively.
