# Supplementary material for: Bacterial Production of Recombinant Coagulation Factor VIII Domains
Source: Medicina (Kaunas). 2023 Apr 1;59(4):694. doi: 10.3390/medicina59040694 (PMC10143837; doi:10.3390/medicina59040694)
Supplement: Supplementary file 1 [file medicina-59-00694-s001.zip › medicina-2267927-supplementary.pdf]

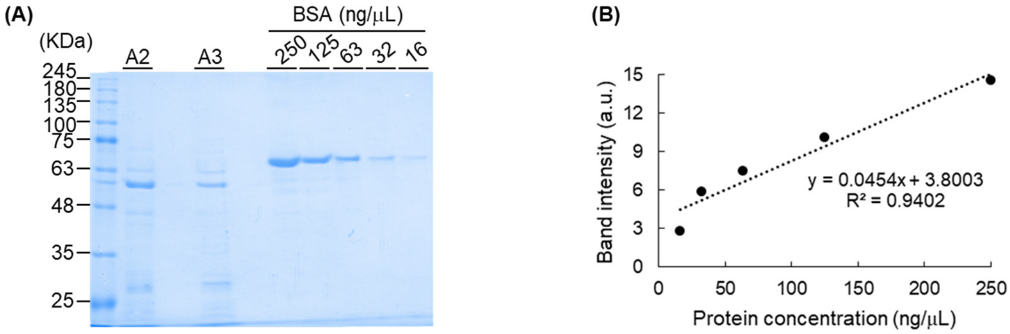

Supplementary Figure S1. (A) SDS-PAGE analysis of GST-conjugated rF8-A2 or rF8-A3 with various concentrations of bovine serum albumin (BSA). 10 μL of recombinant proteins and BSA proteins were loaded. (B) Standard curve for estimating the absolute quantity of target protein. It was calculated that 16.6 μg of GST-A2 and 9.6 μg of GST-A3 were applied on the gel for SDS-PAGE analysis.

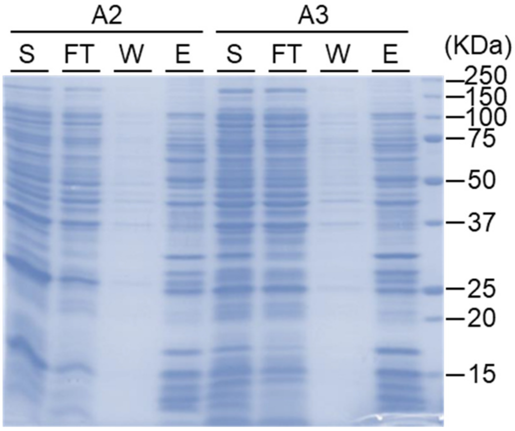

Supplementary Figure S2. SDS-PAGE analysis of the expressed pET28(a)::rF8-A2 or pET28(a)::rF8-A3 proteins. S, FT, W, and E indicates soluble fraction prior to purification, flow through fraction after Ni-NTA bead binding, washed fraction, and eluted fraction, respectively.
